# Pregnancy options counselling in Ghana: a case study of women with unintended pregnancies in Kumasi metropolis, Ghana

**DOI:** 10.1186/s12884-019-2598-7

**Published:** 2019-11-27

**Authors:** Evans Kofi Agbeno, Fred Yao Gbagbo, E. S. K. Morhe, Soale Issah Maltima, Kwadwo Sarbeng

**Affiliations:** 1 Department of Obstetrics and Gynaecology, School of Medical Sciences, University of Cape Coast, Cape Coast, Ghana; 2 Department of Health Administration and Education, Faculty of Science Education, University of Education, Winneba, Winneba, Ghana; 3 Department of Obstetrics and Gynaecology, School of Medical Sciences, University of Allied Sciences, Ho, Ghana; 4 Department of Obstetrics and Gynaecology, School of Medical Sciences, University of Development Studies, Tamale, Ghana; 5 Family Planning Unit, Komfo Anokye Teaching Hospital, Kumasi, Ghana; 6 University of Cape Coast Medical School, Cape Coast, Ghana

**Keywords:** Options counselling, Pregnant women, Unintended pregnancies, Kumasi, Ghana

## Abstract

**Background:**

Pregnancy crisis mismanagement has contributed to maternal deaths and illnesses globally and in Ghana due to absence/inadequate pregnancy options counselling for clients to make informed decisions. This study examines options counselling for abortion seekers in health facilities in Ghana.

**Methods:**

Analytical cross-sectional study design was done in selected specialised public and NGO health facilities within Kumasi Metropolis of Ghana, using self-administered structured questionnaires for data collection from 1st January to 30th April, 2014. Participants were 442 women with unintended pregnancies seeking abortion services. Data was analysed using Epi-Info (7.1.1.14) and STATA 12 to generate descriptive statistics, Pearson chi-square and multivariable logistic regressions. The Kwame Nkrumah University of Science and Technology approved the study.

**Results:**

Respondents had divergent reproductive and socio-demographic profiles. Majority (about 58%) of them had been pregnant more than twice, but about 53% of this population had no biological children. (Although about 90% of respondents held perceptions that the index and previous pregnancies were mistimed/unintended, the majority (72%) had no induced abortion history. Induced abortion (208, 49%) and parenting (216, 51%) were mentioned as the only available options to unintended pregnancy in hospitals. Exposure to options counselling was observed to be significantly associated with parity (*P* **=** < 0.001), gestational age (*P* **=** < 0.001), previous induced abortions (*P* **=** < 0.001), perception of pregnancy at conception (*P* **=** < 0.001) and level of education (*P* **=** 0.002). The logistic regression analysis also shows that higher education has statistically significant effect on being exposed to options counselling (*P* **=** < 0.001). Majority of respondents (95%) were not aware that giving a child up for adoption is an option to abortion in Ghana.

**Conclusions:**

Pregnancy options counselling remains a major challenge in comprehensive abortion care in Ghana. Although higher educational attainments significantly exposes women to options counselling for informed decisions, the less educated are disadvantaged in this regard. Further research on type and depth of counselling services provided to pregnant women in health facilities is required to inform health policy and program decisions.

## Background

Pregnancy options counselling is a form of psychological interaction which provides information and support regarding pregnancy and related issues, often unintended [1]. The ideal counselling often requires an unbiased and non-judgmental approach which allows pregnant women to take their own decision [2]. Such counselling is so important that it influences pregnancy decisions and outcomes [3]. This should therefore not be left for untrained people [4]. Best practice from the British Pregnancy Advisory Service (BPAS) indicates that pregnant women are expected to be allowed to consult widely before and after options counselling and the counselling process requires certain strict competences to be achieved by such counsellors before they can be licensed to handle that job [5].

Counselling is an interactive client beneficial relationship set up to approach a client’s social, cultural and/or emotional issues in a holistic way [6]. Studies have shown that such interactions influence the decisions women make when pregnant [7, 8]. As such when the counselling is either not done or done incorrectly, it increases the extent of regret for the option taken which sometimes has very devastating consequences on the pregnant woman and significant others [9, 10]. This is rightly so particularly in situations where a pregnancy that is wanted but unintended/unplanned occurs has to be aborted because abortion is the only viable option available to the pregnant woman at the time becomes devastating. This is why the current study is very necessary in pregnancy crisis management in Ghana.

Ghana enacted its abortion law [11] in 1960 (PNDC, Law 102; 1960) and developed its comprehensive abortion policy, standards and protocols in 2013 to guide the implementation the law [12]. One of the major components of implementing the comprehensive abortion care policy in Ghana is pregnancy options counselling which has clearly stated national guidelines on the step-by-step approach to counselling [13]. Even though this provision has not given strict guidelines for those who can counsel pregnant women on options, the comprehensive nature of safe abortion care services in Ghana mandates only trained/certified midwives and medical practitioners to do so [14]. Since all providers are taken through a form of training with options counselling as a component, it can be stretched that options counselling training is a requirement of the safe-motherhood package in Ghana. Anecdotal evidence on the increasing incidences of regret following decisions on pregnancy outcomes have however shown that options counselling in pregnancy is a key challenge in some Ghanaian health facilities.

### Aim of the study

The study generally examines the practice of options counselling for abortion seekers in Ghana. Specifically, the proportion of pregnant women exposed to pregnancy options counselling before arriving at a decision with their pregnancies were estimated. The effects of these counselling on decisions relating to pregnancy outcomes and associations between women characteristics and options counselling were also explored to inform Reproductive Health policy and program decision in Ghana.

## Methods

### Study design, population and sampling

This is an analytical cross-sectional study involving first time female visitors with unintended pregnancies seeking pregnancy related healthcare services from the identified health facilities. The use of first time visitors was to help avoid contamination so that no single participant would be recruited more than once. The sample size (462) was calculated using the formula n = pqz^2^/d^2^, where n = sample size, z = level of confidence at 95% =1.96; d = allowable error = 0.05; p = proportion of unintended pregnancies and q = 1-p; p = proportion of unintended pregnancies in Ghana [15] obtained from the 2008 GDHS = 0.4, and thus q = 1–0.4 which translated into 0.6.*n* = 368.7986 which was approximated to 369 plus 25% non-response (the 25% non-response rate was assumed because of the sensitive nature of the subject and the probability of the respondents opting out in the course of the interview as observed during the pretesting).

Purposive sampling method was used to select the health facilities for data collection because they are both situated in the central business area of the city and are well known to provide pregnancy related services in Kumasi. All consenting clients with unintended pregnancy patronising these health facilities were invited into the study until the desired numbers were obtained. In terms of proportions of respondents obtained from the health facilities.

### Study area

This study was conducted in two health facilities [Non-Governmental Organization (NGO) reproductive health center and Public hospital] within the Kumasi Metropolis in Ghana. Kumasi is Ghana’s second biggest city situated about 300 km from the national capital, Accra. The city is 150sq Km in size. Politically, Kumasi is divided into ten sub metropolitan areas namely: Manhyia, Tafo, Suame, Asokwa, Oforikrom, Asawase, Bantama, Kwadaso, Nhyiaeso and Subin. It has been a busy market center and attracts people of varied social background. Most of the people in Kumasi patronize the Adum market and thus facilities in this vicinity are opened to a good mix of clientele involving the Kumasi populace.

The NGO reproductive health center is operated by an international NGO established in 2006 in response to a need for more organizations to deliver sexual and reproductive healthcare in Ghana. The organisation has 2 main health care centres in Kumasi (Adum and Alabar) and also offers reproductive health services through the Blue Star franchise scheme in several private health facilities in Kumasi. The Blue Star is to widen access to safe abortion services while using existing providers to give quality sexual and reproductive health services to clients by strategically selecting shops located in underserved areas in an attempt to reach people who otherwise would not have access to family planning counselling, commodities, and services. One of their main groups of clients is women with unintended pregnancy crisis situation needing help to resolve it.

The Public facility is a Government owned Maternal and Child Health Hospital (MCHH) also located in Adum (the central business district of Kumasi). According to the 2013 Mid-Year Report (unpublished), it has an average of 200 antenatal care new entrants per month. Among these are those with unintended pregnancies requiring professional help for resolution. Its unique location exposes it to a good mix of people from all walks of life.

### Data collection

The data collection started from 1st January-30th April, 2014. Data was collected from consented clients using authors developed structured questionnaire (open and close-ended) after permission was granted by participating facilities. The questionnaire was developed based on information from relevant literature and took a maximum of 15 min to administer. The variables or domains that were collected via the questionnaire include pregnant women’s choice of unintended pregnancy options (independent variable) and exposure to options counselling (dependent variable). Pretesting of the questionnaire was done at a different facility with similar characteristic to ensure validity and reliability of the instrument. Two female research assistants proficient in Ashanti Twi and English were trained and used for data collection. Recruitment of respondents was facilitated by health workers at the respective facilities via clients’ information about the study during the routine general interactions on arrival in the health facilities. Clients were also informed that their participation in the study was voluntary and refusal to take part in the study would not affect the quality of care to be provided. Only clients who voluntarily consented to be part of the study were interviewed using the questionnaires. The questions were read in the language in which a client is most fluent (i.e Twi or English) to ensure clear understanding of what is required. The recruitment of respondents for the interviews were done on a daily basis until the desired numbers were obtained.

### Ethical considerations

The study protocol was registered with the Kwame Nkrumah University of Science and Technology Research and Development Unit to ensure its relevance to the research objectives of the University. The study approval was given by the Committee of Human Research, Publications and Ethics, Kwame Nkrumah University of Science and Technology. Written permissions were sought from the management of facilities in which data was collected. Informed consent and permission to participate in the study were obtained from each respondent. Permission was also sought to have participants’ phone numbers for any follow issues relating to the study.

### Data analysis

Data analysis started with coding, cleaning and entering the data into Epi Info 7 (7.1.1.14). The double entry technique was employed in order to improve the accuracy of the data entry. To ensure confidentiality, questionnaires were put under lock and key and the electronic data was password protected by the principal investigator. In line with the study objectives, data was summarized using frequency distribution and simple proportions for the discrete variables while mean, standard deviation, median, range were used for continuous quantitative variables. Pearson’s chi-square analyses were carried out to test the association between the pregnant women’s choice of unintended pregnancy options (independent variable) and exposure to options counselling (dependent variable). Logistic regressions were also done to observe the relationships between these variables. All computations were done at 95% confidence interval and 5% level of significance (*p* < 0.05).

## Results

Four hundred forty-two (442) pregnant women comprising 227 obtained from the public hospital and 215 from the NGO reproductive health centres participated in the study. Four clients did not complete the interview at the public hospital as they voluntarily withdrew from the study. At the NGO reproductive health centres, 16 of those invited could also not complete the interview. The reason for non-completion of the interview for all the 20 dropouts was time constraints. The overall response rate was 96.1%. Data from women who did not complete the interview were however discarded and not included in the analysis.

Table 1 presents socio-demographic characteristics of respondents. More than half of the respondents were from public facilities with 88.0% aged ≥20. Majority of the respondents (67.2%) are income earners and about 60% of the respondents were never married although pregnant. About 55 had attained at least basic education with Christians recording 89.3% of total respondents. About 67% of the respondents had an average monthly income within the national minimum wage and below.

**Table 1:** Socio-demographic characteristics of respondents

Variables	Frequency	Percentage
Facility of recruitment		
NGO Facility	215	48.6
Public Facility	227	51.4
Total	442	100
Age (in completed year)		
< 20	53	12.0
≥ 20	389	88.0
Total	442	100.0
Occupation		
Income earners	297	67.2
Non-income earners	145	32.8
Total/response rate	442	100.0
Marital Status		
Currently or ever married	176	40.2
Never married	262	59.8
Total	438	100.0
Educational Level		
≤ Basic	244	55.2
> Basic	198	44.8
Total	442	100.0
Religion		
Christian	392	89.3
Muslim	47	10.7
Total	439	100.0
Average monthly income		
National minimum wage and below	239	67.3
Above national minimum wage	116	32.7
Total	355	100.0

Source: Authors field data 2014

The respondents had divergent reproductive profiles (Table 2). About 72% of the respondents had no history of previous induced abortions. Majority of the respondents (about 58%) had been pregnant twice, about 53% had no biological child and about 90% of the respondents held the perception that their pregnancies were mistimed.

**Table 2:** Reproductive profile of respondents

Variable	Frequency	Percentage
Gravidity		
1	188	42.5
≥ 2	254	57.5
Total	442	100.0
Parity		
0(non-parous)	232	52.5
≥ 1(parous)	210	47.5
Total	442	100.0
Gestational Age (in weeks)		
≤ 13	178	50.4
> 13	175	49.6
Total	353	100.0
Previous induced abortions		
Yes	124	28.1
No	318	71.9
Total 442,100.0		
Perception of pregnancy at conception		
Unwanted	44	10.5
Mistimed	377	89.5
Total	421	100.0

Source: Authors field data 2014

**Fig. 1:**
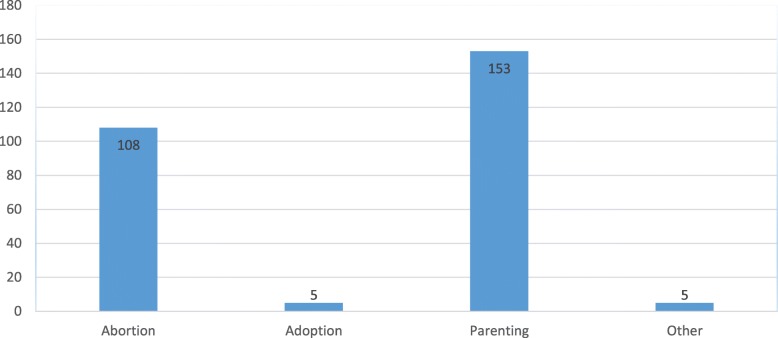
Respondents’ views of possible options to unintended pregnancy prior to visiting hospital (The other options indicated include, baby abonnement, killing or selling after birth)

Prior to visiting a health facility, it was noted that 84.5% of respondents had some interactions with significant others on possible options. The respondents’ awareness of options available for unintended pregnancy following these interactions was therefore ascertained. Induced abortion (108, 40%) and parenting (153, 57%) were the two main responses given as the only know options available to them in the event of unintended pregnancy. Although in minority, the ‘other’ options indicated include, baby abonnement, killing or selling after birth (Fig. 1).

A number of factors were associated with clients’ exposure to options counselling in the facilities visited for services. About (271, 64%) of respondents had exposure to a form of counselling in the facility visited whereas (151, 36%) did not. The significant factors observed include, level of Education *(P < 0.002),* Parity *(P < 0.001),* Gestational Age *(P < 0.001)*, Previous induced abortion *(P < 0.001),* and Perception of pregnancy at conception *(P < 0.001)* (Table 3).

**Table 3:** Factors associated with exposure to options counselling

Factor	Exposure to Options Counseling		*P*- value
	Yes	No	
Facility of recruitment			
Public facility clients	135 (59.5)	92 (40.5)	0.39
NGO facility clients	134 (63.5)	77 (36.5)	
Age category			
< 20 yrs	42 (79.3)	11 (20.8)	0.005
≥ 20 yrs	226 (59.2)	156 (40.8)	
Educational level			
≤ Basic	132 (54.8)	109 (45.2)	0.002
> Basic	137 (69.5)	60 (30.5)	
Occupation			
Non-income earners	99 (70.2)	42 (29.8)	0.008
Income earners	168 (57.0)	127 (43.1)	
Marital status			
Never Married	171 (65.5)	90 (34.5)	0.02
Married	94 (54.3)	79 (45.7)	
Religion			
Christianity	243 (62.5)	146 (37.5)	0.10
Islam	23 (50.0)	23 (50.0)	
Average monthly Income			
≤ minimum wage	147 (62.0)	90 (38.0)	0.36
> minimum wage	66 (57.0)	50 (43.1)	
Gravidity			
1	105 (56.2)	82 (43.9)	0.05
> 1	164 (65.3)	87 (34.7)	
Parity			
Non para	113 (49.8)	114 (50.2)	< 0.001
para 1	154 (73.7)	55 (26.3)	
Gestational Age			
≤ 13 weeks	74 (42.1)	102 (58.0)	< 0.001
> 13 weeks	143 (81.3)	33 (18.8)	
Previous induced abortion			
Yes	232 (73.7)	83 (26.4)	< 0.001
No	37 (30.1)	86 (69.9)	
Perception of pregnancy at conception			
Mistimed	219 (58.1)	158 (41.9)	< 0.001
Unwanted	37 (86.1)	6 (14.0)	

Source: Authors field data 2014

Table 4 presents the logistic regression analysis of factors associated with exposure to options counselling. The results shows that increasing educational levels beyond basic school level education confers statistically significant effect on being exposed to counselling (*p*- value **=** < 0.001). There was almost a seven-fold odds of exposure to counselling when one’s educational level is beyond the basic school level.

**Table 4:** Logistic regression analysis of factors associated with exposure to counselling

Variable	Pregnant women who have had exposure to options counselling (*N* = 271)	Crude odds ratio(95%CI)	Adjusted Odds Ratio(95%CI	*P*- value
Age category				
< 20 yrs	42 (79.3)	Reference	Reference	
≥ 20 yrs	226 (59.2)	0.38 (0.17,0.78)	0.1 (0.05,0.32)	< 0.001
Educational level				
≤ Basic	132 (54.8)	Reference	Reference	
> Basic	137 (69.5)	1.89 (1.25,2.68)	6.82 (2.62,17.72)	< 0.001
Occupation				
Non-income earners	99 (70.2)	Reference	Reference	
Income earners	168 (57.0)	0.98 (0.76,1.26)	0.7 (0.37,1.25)	0.22
Marital status				
Never Married	171 (65.5)	Reference	Reference	
Married	94 (54.3)	0.96 (0.63,1.42)	0.8 (0.47,1.52)	0.58
Gravidity				
1	105 (56.2)	Reference	Reference	
≥ 2	164 (65.3)	1.47 (0.98,2.21)	4.2 (1.48,11.68)	0.01
Parity				
0	113 (49.8)	Reference	Reference	
≥ 1	154 (73.7)	2.82 (1.85,4.32)	2.74 (1.12,6.70)	0.03
Gestational Age				
≤ 13 weeks	74 (42.1)	Reference	Reference	
> 13 weeks	143 (81.3)	5.97 (3.59,10.0)	2.3 (1.29,3.95	0.004
Previous induced abortion				
No	37 (30.1)	Reference	Reference	
Yes	232 (73.7)	6.5 (4.01,10.59)	3.3 (1.33,8.24)	0.01
Perception of pregnancy at conception				
Mistimed	219 (58.1)	Reference	Reference	
Unwanted	37 (86.1)	4.45 (1.8,13.170	6.93 (2.14,22.46)	0.001

Source: Authors field data 2014

## Discussions

One of the primary objectives of this study was to examine the proportion of respondents exposed to options counselling before they arrived at a decision on their pregnancy. About (271, 64%) had exposure to a form of counselling whereas (151, 36%) did not. Noteworthy is the fact 84.5% had interactions with significant others before visiting the health facility. In terms of proportion exposed to options counselling, the respondents who were recruited from the NGO facilities appeared to be slightly higher compared to those from the public, although not statistically significant. It would have been expected that all clients would have been aware of options counselling because all the respondents had gone through the processes at both public and NGO health facilities (where counselling is supposed to be part of the care plan) before the interviews were conducted. Could it have been that some respondents went to health facilities having already made up their minds on what they wanted to do with their pregnancies or were they actually not exposed to options counselling as being reflected in the response from the study?

To assess the depth of counselling participants were exposed to; their awareness of the options for the unintended pregnancies was used. The multiple response data set used allowed the respondents to mention all the options they knew. The fact that the awareness of both abortion and parenting was high (40 and 57% respectively) was not surprising but the finding that only 1.9% of the respondents knew of adoption as an option was. This is because all of the respondents were either Christians or Muslims whose religions encourage adoption [16, 17] and interestingly, about the same percentage mentioned ‘throwing the baby away after delivery’ and selling the child to interested parties as options. It stands to reason that there is a high probability that the options counselling given at the facility is not adequate. In Ghana where there appears to be increasing advocacy on women’s right to reproductive options including choices on pregnancy outcomes, the over emphasis on induced abortion from programmes stance and parenting from religious point of view creates an environment where other options to unintended pregnancies become limited. There is therefore a need to intensify public education on the legal options and available services, available to women carrying unintended pregnancies at any gestational age to make informed choices. In an abortion study in Ghana [18], it was noted that clarifying the roles and responsibilities of primary care clinicians and providing them with state-of-the-art tools and training in the management of unintended pregnancy is equally important for solving this public health challenge. Another study recommended the need to establish culturally appropriate evidence and competency based clinical guidelines for the prevention and management of unintended pregnancy that can be integrated into primary care and the broader health system and that are built on a comprehensive public health framework for pregnancy prevention that specifies the essential competencies required of all members of the health care team [19].

In terms of proportion, the 20 years and above respondents appeared to have been less predisposed to getting counselled and the multivariate analysis supported that observation since the odds of exposure to counselling for them was 0.1,95%CI:0.05–0.32 and was statistically significant (*p* < 0.001). It can therefore be said that as one ages, there is less consultation with others in an event of unintended pregnancy. Those in their teens are more likely to access help from others as this might have been certainly a new area in their growth process and require the guidance of significant others. This finding is consistent with those of other studies on decision making for abortion [8, 20].

Education has been found to play a vital role in abortion decision-making [8, 21]. In this current study, it was noted that increasing education level beyond the basic education confers statistically significant effect on being exposed to counselling. There was almost a seven-fold odds of exposure to counselling when one’s educational level is beyond the basic level. Increasing access to education beyond the basic level is vital for some level of consultation before unintended pregnancy decisions are made. Thus, access to options counselling can be increased through the School Health Programme. This will invariably lead to a better outcome for the unintended pregnancy crisis management and eventually reduce child abandonment, unsafe abortion maternal and neonatal deaths and illnesses.

Occupation as categorized into income earnings did not seem to have statistically significant influence on exposure to counselling even though in terms of proportion, non-income earners were more likely to be exposed to counselling since they may be less economically empowered to deal with the implications of each of the unintended pregnancy outcomes. In the same vein, the actual earnings (in terms of the national minimum wage of 4.6 Ghana cedis, 2013, which was still in use at the time of data collection), also failed to support any predisposition to counselling exposure. In a related study [22], it was observed that disclosers or nondisclosure of unintended pregnancies did not differ in occupational levels of women seeking abortion services. This shows that where a woman is desperately in need of an abortion service, the level of education does not really matter on options counselling since such women would have already made up their minds on what is best for them at the time with an aim to secure their occupations.

It was being expected that first time pregnancies would rather make more people confused when it was not anticipated and may require significant others to make their decision. Instead, the study found that being pregnant more than once rather increased the exposure to counselling by more than four folds. This observation appears to logically infer that the decisions made on previous pregnancies outcomes might have influenced the current hence need to tread more cautiously by seeking the support of significant others to resolve any pregnancy related challenges. Findings from a study in Holland [23], for example, shows that following the pill panic in the early 1980s women who visited reproductive health centres reported that they did not know they could get pregnant the first time they had unprotected sexual intercourse and subsequently took contraception counselling more serious as an option to prevent occurrence of future unplanned pregnancies. In the Ghanaian context the introduction of sexuality education in the educational system of Ghana with a counselling component seems to be in the right direction as it will enable young people in school to make informed choices.

Birthing experience also increased respondents’ exposure to counselling by almost three folds. This trend appears to have supported the observations made for gravidity. One other reason for both parous and multigravida women to go for counselling might have been their previous encounter with the health system through their previous pregnancies and can thus more readily locate such resources.

The respondents were about twice as likely to be exposed to counselling, when the pregnancy had travelled past the first trimester, than those within the first trimester. This might be as a result of the women’s initial inability to resolve the pregnancy crisis on their own and needed others to help them. It appears the older a pregnancy gets, the more difficult it is for the women themselves to take an independent decision and therefore resort to others to help resolve it. When juxtapose with the average reporting time of 16 weeks, it presupposes that there is the need to give pregnant women more access to quality counselling to bring the best outcomes for the unintended pregnancies.

The study also found that when the perception of pregnancy at conception was in the unwanted category, respondents had an almost seven fold predisposition to receiving counselling compared to those in the mistimed category. It is logical to infer that because respondents do not require having more babies at the time of conception, they were in a more confused state than those with mistimed pregnancies and thus needed significant others to help them resolve the challenges associated with unwanted pregnancies.

All the other socio-demographic and reproductive characteristics of respondents examined did not show any statistically significant associations with options counselling for pregnancy. This observation implies that the health facilities where clients visited at the onset of their unintended pregnancies did not extensively counsel them on all the options available in resolving pregnancy related crises. Also, religion, marital status and average monthly income did not expose them to receiving the required options counselling for unwanted pregnancy. This observation is consistent with the general practice of using induced abortion as a money making venture by some health practitioners. For instance, a former Medical Officer at the Eastern Regional Health Directorate in Ghana, Dr. Joe Taylor once described the pelvis of women as a ‘goldmine’ where quack healthcare providers are busily making huge sums of money through galamsey (illegal mining) or unsafe abortion [24]. In such situations, options counselling is not really a priority in most facilities that provide abortion services. Rather the focus is to have abortions done on demand and even increase the number of services by setting targets for providers in some reported facilities.

## Conclusions

This study examined the proportion of pregnant women exposed to pregnancy options counselling before making decisions with their pregnancies, the effect of these counselling on decisions relating to pregnancy outcomes and the association between women characteristics and pregnancy options counselling. The observation that pregnancy options counselling positively influences decisions on pregnancy outcomes is good and must be encourage. However, the practice as observed in this study is not adequate in health facilities and this makes it imperative for the Ministry of Health and the Ghana Health Service to adopt a multisector approach by involving the Cultural Ministry, the Social Welfare Department and the religious and traditional leadership to establish a non-biased/judgemental, health professional lead one-stop facility at the community level to help people faced with challenges associated with unintended pregnancies resolve it professionally and safely.

Options counselling in pregnancy remains a major challenge in some Ghanaian health facilities as evident by the high number of pregnant women seeking abortion after 13 weeks. This observation calls for early education on pregnancy options for better outcomes. The study also recommends further research using a mixed method approach to assess the type and depth of counselling services provided for pregnant women in health facilities to inform reproductive health policy and programme decisions.

### Study limitations

The study focused purposively on the state of options counselling in public and NGO facilities. The private sector being the largest abortion providers in Ghana was not included in the study due to available evidence that they lack relevant systems, structures and dedicated counsellors who could be studied. This is likely to skew the findings to one directions. A comparative study is therefore recommended to examine the topic across the public, private and NGO facilities in Ghana for a border view for comprehensive and holistic interventions.

## Data Availability

The raw data and any material related to the study is available upon reasonable request.

## Abbreviations

ANC: Antenatal care
GDHS: Ghana Demographic and Health Survey
NGO: Non-Governmental Organisation
WHO: World Health Organization
